# Gastrointestinal tract pathology of the owl monkey (*Aotus* spp.)

**DOI:** 10.1177/03009858231204260

**Published:** 2023-10-13

**Authors:** Martha E. Hensel, Aline Rodrigues-Hoffmann, Beth K. Dray, Gregory K. Wilkerson, Wally B. Baze, Sarah Sulkosky, Carolyn L. Hodo

**Affiliations:** 1 The University of Texas MD Anderson Cancer Center, Bastrop, TX; 2 University of Florida, Gainesville, FL; 3 Charles River Laboratories, Ashland, OH; 4 North Carolina State University, Raleigh, NC; 5 The Joint Pathology Center, Silver Spring, MD

**Keywords:** *Aotus* spp., gastrointestinal, intestinal incarceration, owl monkey

## Abstract

Owl monkeys are small nocturnal new world primates in the genus *Aotus* that are most used in biomedical research for malaria. Cardiomyopathy and nephropathy are well-described common diseases contributing to their morbidity and mortality; less is known about lesions affecting the gastrointestinal tract. Records from a 14-year period (2008-2022) at the Keeling Center for Comparative Medicine and Research were queried to identify instances of spontaneous gastrointestinal disease that directly contributed to the cause of death from the 235 adult owl monkeys submitted for necropsy. Of the 235, 10.6% (25/235) had gastrointestinal disease listed as a significant factor that contributed to morbidity and mortality. Diagnoses included candidiasis (3/25), gastric bloat (4/25), and intestinal incarceration and ischemia secondary (11/25), which included intussusception (4/25), mesenteric rent (3/25), strangulating lipoma (2/25), intestinal torsion (1/25), and an inguinal hernia (1/25). Intestinal adenocarcinomas affecting the jejunum (4/25) were the most common neoplasia diagnosis. Oral squamous cell carcinoma (1/25) and intestinal lymphoma (2/25) were also diagnosed. This report provides evidence of spontaneous lesions in the species that contribute to morbidity and mortality.

Nonhuman primates (NHPs) in the genus *Aotus* (common name owl monkeys) are a small (800-1100 g) nocturnal primate species that originates from South America.^49,12^ Owl monkeys were originally considered one species, *A. trivirgatis*, but phylogenetic studies have now separated the genus into 11 species.^42^ *Aotus* spp. have been used as animal models primarily for malaria research, but the full potential of this species has not been explored.^24^

As with many NHP species, a limitation to husbandry and research with this species is a dearth of knowledge about background diseases. The most common and well-characterized spontaneous lesions reported in this species are cardiomyopathy, aortic dissection, and glomerulonephritis.^1,16,17,26,42^ Glomerulonephritis, both spontaneous and associated with *Plasmodium* sp. infection, is perhaps the most investigated disease in owl monkeys.^8,26^ However, less is reported about conditions affecting the gastrointestinal tract, which represents a significant gap in knowledge of conditions that likely contribute to morbidity and mortality within a colony. A recently published comprehensive review of the literature identified 3 lesions of the gastrointestinal tract including herpesvirus lingual ulcers, necroulcerative colitis associated with *Yersinia pseudotuberculosis*, and bacterial catarrhal enteritis.^42^ To address this knowledge gap, diagnostic cases in *Aotus* spp. from 2008 to 2022 submitted for necropsy were reviewed for gastrointestinal tract lesions.

## Materials and Methods

### Animals and Tissue Sampling

Animals were housed within the Association for Assessment and Accreditation of Laboratory Animal Care accredited facilities at The University of Texas, MD Anderson Cancer Center, Keeling Center for Comparative Medicine and Research in Bastrop, TX. All husbandry and necropsy procedures associated with these animals were approved by the Institutional Animal Care and Use Committee at The University of Texas MD Anderson Cancer Center and followed standards established by the Guide for the Care and Use of Laboratory Animals.^36^ The Keeling Center for Comparative Medicine and Research houses 5 species of owl monkeys including *Aotus nancymaae*, *A. vociferans*, *A. azarae*, *A. lemurinus griseimembra*, and hybrids. During a 14-year period (2008-2022), 235 adult owl monkeys (greater than or equal to 2 years of age) were euthanized and submitted for a complete necropsy.^12^ Animals included in the study had not been assigned to experimental protocols and were naïve. Cases were reviewed for causes of mortality and were included if gastrointestinal tract disease was considered a significant contributing factor to morbidity and mortality. Animals less than 1 year of age were excluded from the search due to limited archival information available for this cohort.

Tissues were collected according to the institutional standard operating procedures in 10% neutral buffered formalin and were routinely processed, embedded, and stained with hematoxylin and eosin or other special stains, as needed. Necropsies and histologic evaluations were performed by board-certified veterinary anatomic pathologists. A single case was referred to the Armed Forces Institute of Pathology [Joint Pathology Conference] for immunohistochemistry for CD3 (prediluted clone 2GV6, Ventana Medical Systems), CD79a (prediluted clone L26, Ventana Medical Systems), and CD56 (1:100; clone MRQ-42, Cell Marque).

Paraffin scrolls from 3 cases were submitted for panfungal polymerase chain reaction (PCR) at the Molecular Fungal ID Lab, University of Florida. Briefly, DNA was extracted using the QIAmp DNA FFPE Advanced kit (Qiagen), following the manufacturer’s protocol. PCR targeting the *internal transcribed spacer region 2* and the *large ribosomal subunit* was performed on the extracted DNA, as previously described.^23^ PCR products were run on a 2% agarose gel, and bands were excised from the gel and submitted for Sanger sequencing. The resulting sequences and contigs were analyzed with the NCBI Blast database.

## Results

### Study Population

Signalment information for the entire sample population is listed in Table 1. Animals ranged in age from 2 to 30 years old, with 172 *Aotus nancymaae*, 31 *A. vociferans*, 19 *A. azarae*, 11 *A. lemurinus griseimembra*, and 2 hybrids (Table 1). Of these 235, 25 (10.6%) were diagnosed with gastrointestinal disease at necropsy. The most common diagnoses are listed in Table 2.

**Table 1.** Species and sex distribution of *Aotus* spp. submitted for necropsy from 2008 to 2022.

Species	Total	Males	Females
*Aotus nancymae*	172	76	96
*Aotus azarae*	19	4	15
*Aotus l. griseimembra*	11	9	2
Aotus vociferans	31	18	13
Hybrids	2	0	2

**Table 2.** Infectious, degenerative, and neoplastic conditions identified in the Keeling Center for Comparative Medicine and Research population of owl monkeys.

Diagnosis	Number Affected	Species	Sex	Median Age (Years)
Infectious				
Candidiasis	3	AN (2) AV (1)	M (2) F (1)	4
Degenerative				
Gastric bloat	4	AN (3) AA (1)	M (2) F (2)	4
Intussusception	4	AN (4)	M (1) F (3)	10
Mesenteric rent	3	AN (3)	M (2) F (1)	5
Strangulating lipoma	2	AN (1) AV (1)	F (2)	4.5
Intestinal torsion	1	AV	M (1)	9
Inguinal hernia	1	AV	M (1)	9
Neoplastic				
Adenocarcinoma	4	AN (3) AV (1)	M (3) F (1)	10.5
Lymphoma	2	AA (1) AN (1)	M (1) F (1)	16.5
Oral squamous Cell carcinoma	1	AA	F	7

Abbreviations: AN, *Aotus nancymae*; AV, *A. vociferans*; AA, *A. azarae*; F, female; M, male.

### Mycotic Glossitis, Esophagitis, and Gastroenteritis

**Figure 1.**
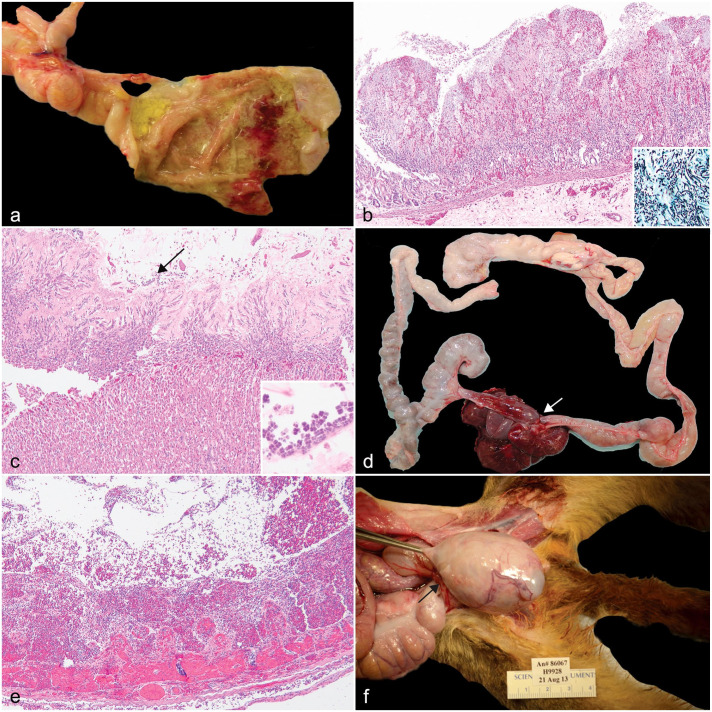
Gastrointestinal tract pathology in owl monkeys. (a) Multifocal ulcerative and hemorrhagic gastritis, stomach, *Aotus nancymaae*. (b) Focal necrohemorrhagic gastritis with invasive hyphae, pseudohyphae, and yeasts. Stomach, *A. nancymaae*. Hematoxylin and eosin (HE). Inset: stomach, 2–4 µm diameter hyphae, pseudohyphae, and yeasts. Grocott’s methenamine silver. (c) Catarrhal gastritis with intralesional *Sarcina* sp. (arrow), stomach, *A. nancymaae*. HE. Inset: high magnification of tetrads of *Sarcina* sp. HE. (d) Strangulating lipoma (arrow) with entrapped jejunum and ileum, *Aotus nancymaae*. (e) Entrapped intestine from a strangulating lipoma, transmural hemorrhage and necrosis, jejunum, *A. nancymaae*. HE. (f) Inguinal hernia (arrow) with entrapped cecum that compresses the testis, *A. vociferans*.

Three animals ranging in age from 2 to 5 years old were diagnosed with fungal infections affecting the oral cavity, esophagus, stomach, and/or intestines. All affected animals presented with anorexia, weight loss, and lethargy. Two animals, a 2-year-old *A. vociferans* and a 5-year-old *Aotus nancymaae*, were diagnosed with oral mycosis primarily affecting the tongue and esophagus. Gross lesions consisted of mucosa thickened up to 4 mm by tan to yellow plaques, which was characterized histologically by hyperplastic epithelium with parakeratotic crust containing mats of invasive yeasts and 2 to 4 µm diameter hyphae. A 4-year-old *Aotus nancymaae* was diagnosed with gastrointestinal mycosis with lesions localized to the stomach (Fig. 1a) and ileum. Histologically, the mucosa was multifocally necrotic and ulcerated with a marked inflammatory infiltrate composed of macrophages, neutrophils, and fewer lymphocytes and plasma cells. Admixed within the necrotic debris and fibrin were mats of invasive 2 to 4 µm diameter fungal hyphae, pseudohyphae, and yeasts (Fig. 1b). Special staining with Grocott’s methenamine silver stain revealed myriad fungal hyphae and yeasts within the ulcerated mucosa (Fig. 1b, inset).

Panfungal PCR targeting the *internal transcribed spacer region 2* and *large ribosomal subunit* yielded poor quality DNA from all 3 cases that could not be used to speciate the fungal organisms or that matched contaminants within PCR reagents (*Malassezia* sp.). It is possible that inhibitors within the paraffin block or resident microbiota from the oral cavity interfered with sequencing attempts.^32^ However, based on the morphology, *Candida* sp. were considered the most likely suspects.

### Gastric Bloat

Gastric bloat was diagnosed in 3 *A. nancymaae* and one *A. azarae* (4/25, 16%) ranging in age from 3 to 9 years old with a median of 4 years old (Table 2). The owl monkeys died acutely with no premonitory clinical signs. Upon gross examination, the stomachs were gas-distended, and the mucosa was congested. Additionally, the abdominal cavity contained 5 to 20 mL of serosanguinous effusion. Histologically, the mucosa was multifocally eroded with thickening of the mucus layer containing numerous 1 to 2 µm tetrad clusters of bacteria interpreted as *Sarcina* sp. (Fig. 1c).

### Intestinal Incarceration and Ischemic Injury

The most common gastrointestinal lesion in the owl monkey population was intestinal entrapment with ischemic injury (11/25, 44%), which encompasses several manifestations including intussusception, mesenteric rent with intestinal entrapment, strangulating lipoma, intestinal torsion, and inguinal herniation. Regardless of the inciting cause, the histologic features of the entrapped intestine were similar: mucosal necrosis with neutrophilic infiltrate and transmural hemorrhage and edema.

Intussusception was diagnosed in 4 *A. nancymaae* in the mid-jejunum (enteroenteric) and descending colon (colocolic). The median age at diagnosis was 11 years, with a range of 9 to 12 years. In 2 animals, the intussusception was characterized by approximately 6 to 8 cm of the distal colon that telescoped into the rectum and protruded 1 cm out of the anus resulting in rectal prolapse, hemorrhage, and acute morbidity. Intussusception led to intestinal perforation and septic peritonitis characterized by fibrinous effusion with intestinal contents in the 10-year-old *Aotus nancymaae* male. Clinical workup for this case included a complete blood count, which revealed a mild, normocytic, normochromic, regenerative anemia (28% hematocrit [ normal range: 49%-52%]) consistent with acute hemorrhage. Regardless of the location of the intussusception, the histologic features were similar: the intussuscipiens had transmural necrosis with hemorrhagic contents.

Mesenteric rent was diagnosed in 3 *Aotus nancymaae* with a median age of 5 years. All animals were found deceased or moribund with no premonitory clinical signs. At necropsy, the gross lesions were similar between the 3 cases. A defect in the mesentery contained entrapped small intestine (n = 2) or small intestine, cecum, and colon (n = 1). The entrapped intestines were gas distended, friable, and the mucosa was dark red to black with hemorrhagic contents.

One *A. nancymaae* and one *A. vociferans* (median age of 4.5 years) were diagnosed with strangulating lipomas. In both cases, the animals had no demonstrable clinical signs but were found deceased. The abdominal cavity contained approximately 5 to 10 mL of serosanguinous fluid. In the 3-year-old, a band of tissue with a 1 cm by 0.5 cm by 0.5 cm lipoma encircled the proximal ileum and mid to distal jejunum approximately 5 cm proximal to the cecum (Fig. 1d); the wall of the incarcerated intestine had transmural necrosis with hemorrhage (Fig. 1e). The duodenum and proximal jejunum aborad to the torsion were gas distended. In the 9-year-old, the stalk of the lipoma extended from the greater omentum and encircled the entire ileum from the base of the cecum to the jejunum.

Intestinal torsion was diagnosed in a 9-year-old male *Aotus nancymaae*, with an approximately 3-year clinical history of weight loss that was unresponsive to medical intervention. A diagnostic biopsy was obtained from the small intestine under general anesthesia; 3 days later, the animal experienced vomiting and collapse. Supportive therapy was attempted to correct dehydration and hypoglycemia, but the animal died. At necropsy, an 8 cm by 3 cm mass of coiled, tightly adhered jejunal loops were found approximately 8 cm distal to the stomach. The intestine aborad to the intestinal torsion was dilated, and the serosal surface was edematous with petechiae. The intestinal biopsy site may have formed adhesions leading to ileus and torsion.

A 9-year-old male *A. vociferans* was diagnosed clinically with a nonreducible 6 cm by 4 cm by 3 cm mass in the right inguinal area. Surgical correction was not successful, and humane euthanasia was elected. At necropsy, the hernia wall was thin and the sac contained clear fluid and approximately 7 cm of entrapped cecum that displaced and compressed the right testis (Fig. 1f).

### Neoplasia

**Figure 2.**
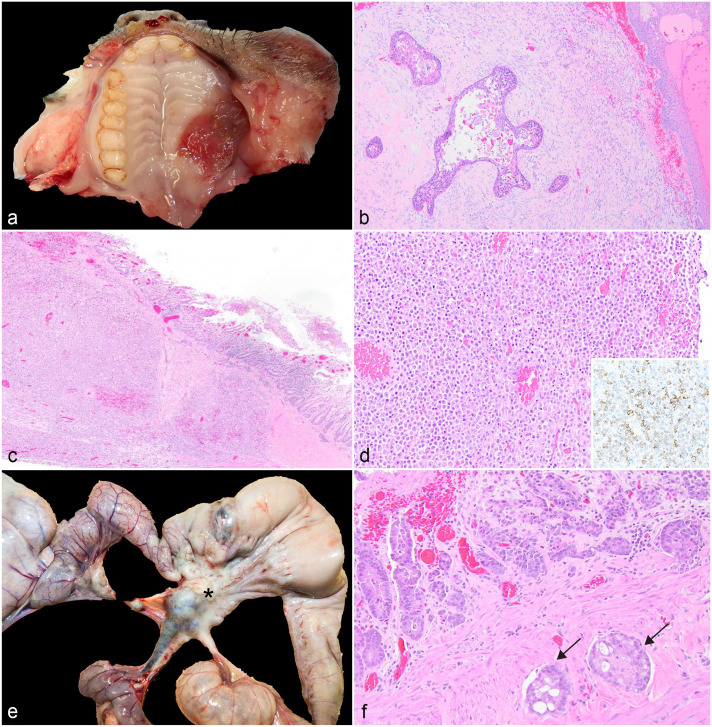
Gastrointestinal neoplasia in owl monkeys. (a) Oral squamous cell carcinoma, hard palate, *Aotus azarae*. (b) Neoplastic cells are arranged in nests with keratinizing epithelium surrounded by a scirrhous stroma, *A. azarae*. Hematoxylin and eosin (HE). (c) An infiltrative population of neoplastic lymphocytes extends from the mucosa of the jejunum to the serosa, *A. nancymaae.* HE. (d) Neoplastic lymphocytes are large round cells with round nuclei. Neoplastic cells have strong membranous labeling with CD3 confirming the T cell origin (inset, CD3 immunohistochemistry). (e) Intestinal adenocarcinoma (*) with metastasis to mesenteric lymph nodes, jejunum, *Aotus nancymaae*. (f) Neoplastic epithelial cells are arranged in nests and tubules. The mucosa is eroded with hemorrhage. Multiple vessels contain intravascular islands of neoplastic cells (arrows). HE.

Oral squamous cell carcinoma was diagnosed in a 7-year-old female *A. azarae* (Fig. 2a, b). The neoplasm was locally aggressive and invaded the hard palate, palatine bone, and orbit and tracked along the optic nerve. Clinically, the animal had exophthalmos and epiphora from the neoplastic invasion of the retro-orbital space. The mandibular lymph node was enlarged, and neoplastic cells were histologically identified in the lymph node. Additionally, intravascular metastases were identified in the lung.

Intestinal neoplasia was diagnosed in 6 owl monkeys with a median age of 13 years with a range of 9 to 17 years. Regardless of the diagnosis, the clinical signs were non-specific: weight loss and lethargy.

Lymphoma was diagnosed in the mid-jejunum of 2 adults based on histologic features of an infiltrative round cell population (Fig. 2c, d) and immunohistochemical staining properties. In both cases, the jejunal wall was thickened and formed pseudodiverticula with an ulcerated mucosal surface covered by a black-to-green plaque of necrotic debris and fibrin, and the mesenteric lymph nodes were diffusely enlarged with a loss of the corticomedullary distinction. Histology confirmed lymphoma in the mesenteric lymph nodes. Immunohistochemistry for CD3 performed at the Joint Pathology Conference confirmed the T cell origin in one case (Fig. 2d, inset). In the second case, immunohistochemistry was inconclusive due to prolonged post-mortem interval.

Small intestinal adenocarcinomas were diagnosed in 4 adults ranging in age from 9 to 15 years old. Intestinal adenocarcinomas were characterized by concentric “napkin-ring,” firm, tan masses in the proximal jejunum (n = 3) and ileocecal junction (n = 1) with dilation of the aborad intestine (Fig. 2e). Neoplastic epithelial cells formed nests and tubules on a desmoplastic stroma and had invaded the vasculature (Fig. 2f). Vascular invasion was noted in all cases with metastases to the mesenteric lymph nodes. In addition, one case had abdominal carcinomatosis affecting the mesentery and omentum, and metastasis to the liver and lungs.

## Discussion

In the 14-year period examined, 10.6% (n = 25) of the owl monkeys submitted for necropsy were diagnosed with gastrointestinal disease. Of these, the most common diagnoses were gastric bloat (16%), intestinal incarcerations with ischemic necrosis (44%), and intestinal adenocarcinoma (16%).

Infectious causes of gastroenteritis were uncommon and did not include previously reported conditions such as *Yersinia*-associated typhlocolitis or herpesvirus lingual ulcers.^42^ The only infectious cause identified in the owl monkeys was lingual, esophageal, and gastrointestinal candidiasis. Oral mycosis has been reported in several NHP species, including *Aotus* sp., and is often associated with immunosuppression or prolonged antibiotic therapy.^33,42,44,49^ *Candida* sp. can be cultured from apparently healthy animals and humans and is considered part of the normal mycobiota.^30,40,46^ However, when the balance of the resident microbiome is disrupted, the yeasts can become invasive hyphae and lead to ulceration and inflammation.^30,46^ In the cases presented herein, one animal had abdominal exploratory surgery to remove a kidney with neoplasia and subsequently decompensated. The other 2 had diarrhea of undetermined cause with significant weight loss of approximately 200 g over a 66-month time course, which was treated empirically with supportive care and antibiotics.

Gastric bloat is one of the most commonly diagnosed causes of gastrointestinal disease in owl monkeys.^6,17,49^ A review of gastric bloat in nonhuman primates indicate that gastric bloat is accompanied by hypovolemic shock, acid-base disturbances, and electrolyte derangements that may affect cardiac function and lead to acute mortality.^38^ Bloat has been reported in several species of NHP including macaques (*Macaca* spp.), squirrel monkeys (*Saimiri* spp.), spider monkey (*Ateles geoffroyi*), chimpanzees (*Pan troglodytes*), and baboons (*Papio* spp).^11,29,38,42^ The underlying pathogenesis is not well defined, but dietary factors such as over-eating, microbial fermentation of readily fermentable food, disrupted feeding schedules, and fasting for anesthesia have all been suggested as predisposing factors.^38^ In this report, 3 of 4 animals with gastric bloat also had intralesional *Sarcina* sp. within areas of inflammation and catarrhal exudate. In domestic species such as goats and cats, *Sarcina ventriculi* has been associated with delayed gastric emptying and bloat.^9,27^ The association in NHPs is speculative as *Sarcina* sp. can be part of the normal flora, and overgrowth may represent dysbiosis.^29^ However, a report on chimpanzees at a sanctuary describes a newly discovered *Sarcina* sp. associated with an epizootic neurologic and gastroenteric syndrome characterized clinically by gastric bloat and pain.^37^

Intestinal incarcerations with ischemic necrosis occurred in 44% of the population with gastrointestinal tract pathology. Intussusception in NHP species is more often reported in young animals and may be associated with diarrhea or decreased intestinal motility.^11,29^ Intussusceptions may occur spontaneously but have also been diagnosed secondary to parasite infestations with *Gastrodiscoides hominis* in a rhesus macaque or *Trichuris* sp. in 2 juvenile baboons (*Papio hamadryas*).^14,22^ The owl monkeys had no evidence of intestinal parasites and were part of a closed colony housed indoors. Interestingly, intussusception in humans is often diagnosed in children from 3 months to 6 years of age, and the clinical symptoms include vomiting, diarrhea, abdominal pain, and hematochezia.^48^ In contrast to the locations of mid-jejunum or distal colon noted in these owl monkeys, Meckel’s diverticulum is the most common location for intussusception in children.^48^

Mesenteric rent with intestinal entrapment has not been described as a spontaneous lesion in *Aotus* sp. or other NHP species. This condition is a well-known cause of intestinal incarceration in equids, but the root cause is often speculative. Proposed underlying mechanisms include congenital defects, trauma, or intestinal masses that exert a tearing force on the mesentery.^15^ The margins of the mesenteric defects in the owl monkeys were smooth, which suggests either a chronic tear from trauma or a congenital lesion.

Intestinal torsion has not been reported in owl monkeys. In rhesus, colonic volvulus may occur secondary to abdominal surgery that forms adhesions, diarrhea, megacolon, sedentary lifestyle, or atherogenic diets.^28^ The clinical signs are often vague and include abdominal distension and shock, which may not be detected during routine evaluation.^28^ In this case, the torsion is likely secondary to previous abdominal surgery to obtain intestinal biopsies because the small intestine was twisted around adhesions that formed at the surgical site.

Inguinal hernias have been reported in several species of macaques including rhesus (*M. mulatta*), cynomolgus (*M. fascicularis*), pig-tailed (*M. nemestrina*), and bonnet (*M. radiata*).^7,13,39,47,50^ This condition is not reported in the *Aotus* spp. literature and is a rare diagnosis in the colony. In the medical literature, inguinal hernias are more common in males than females, and the pathogenesis involves congenital and acquired components.^20^ In patients with inguinal hernias, the majority are thought to have a congenital defect in the closure of the processus vaginalis, which perpetuates a peritoneal opening at the inguinal ring.^20^ A recent review of inguinal hernias in NHP species noted a similar sex predisposition toward males; the pathogenesis likely involves a congenital defect that develops into a clinically significant disease secondary to obesity, straining, or long-term cough.^10^ Approximately 800,000 people per year have surgery to correct inguinal hernias either through mesh or primary repair; an attempt was made to surgically reduce the hernia in the owl monkey but was unsuccessful.^20^

Squamous cell carcinoma of the oral cavity has been identified in other NHP species, including several species of macaque, squirrel monkey, baboon, capuchin monkey (*Cebus apella*), common marmoset (*Callithrix jacchus*), and a spider monkey (*Ateles* spp.).^4,18,19,43,45^ This is the first report of this tumor in *Aotus* spp. The pathogenesis is not well defined in NHP, and genetic markers or predisposing conditions are an area in need of research.^19^

T-cell lymphoma can be spontaneous or associated with long-term infection with herpesvirus saimiri.^2,25,31,34^ Herpesvirus saimiri is a *Rhadinovirus*, and T cells appear to be the target of oncogenic transformation.^34^ Transmission requires contact between infected squirrel monkeys and owl monkeys and results in long-term disease. In squirrel monkeys, the virus can be horizontally transmitted without causing clinical disease.^25^ When an owl monkey is infected via experimental inoculation or through contact with a carrier, long-term infection of T cells can lead to lymphoproliferative disease.^2,31,34^ We cannot rule out the possibility of herpesvirus saimiri because tissue was not available for testing in the first case and in the second case, the tissues were considered too autolyzed to attempt ancillary testing. However, the cases presented herein were considered spontaneous due to institutional biosecurity protocols in place to prevent transmission between species and the rare nature of the diagnosis.

Intestinal adenocarcinoma is a common diagnosis in older NHPs such as rhesus macaques, squirrel monkeys, and common marmosets, but it has not been previously reported in *Aotus* spp.^5,29,35^ Small intestinal adenocarcinomas are less frequent than colorectal carcinomas in humans, but the incidence appears to be increasing.^3^ The primary location of the tumors in *Aotus* spp. was the jejunum. The location is similar to that reported for common marmosets but is in contrast to rhesus macaques, which are commonly located at the ileocecal junction or colon.^21,35,41^ All of the adenocarcinomas in the owl monkeys metastasized to the mesenteric lymph nodes, while carcinomatosis and metastasis to the liver were identified in a single case; metastasis is reported in approximately 30% of rhesus macaques diagnosed with colon carcinoma.^41^

## Conclusion

In summary, we have described several instances of previously unreported conditions affecting the gastrointestinal tract in *Aotus* spp., including intestinal incarcerations and intestinal adenocarcinomas. Gastrointestinal disease is not a frequent cause of death but should be considered for moribund or unexpected deaths. This report provides an overview of gastrointestinal disease in the species, and intestinal carcinoma could also represent a new area of research interest.
